# Corporate Social Responsibility and Cheating Behavior: The Mediating Effects of Organizational Identification and Perceived Supervisor Moral Decoupling

**DOI:** 10.3389/fpsyg.2021.768293

**Published:** 2022-01-04

**Authors:** Kun Luan, Mengna Lv, Haidong Zheng

**Affiliations:** ^1^School of Economics and Management, China University of Petroleum (East China), Qingdao, China; ^2^Business School, Central University of Finance and Economics, Beijing, China

**Keywords:** corporate social responsibility (CSR), cheating, organizational identification, perceived supervisor moral decoupling, employee bottom-line mentality

## Abstract

Previous corporate social responsibility (CSR) studies at the employee level have focused on the influence of CSR on employees’ positive attitudes and behavior. However, little attention has been paid to the relationship between CSR and unethical behavior and the underlying mechanism. Based on social information processing theory, this study investigates how CSR affects employee cheating *via* employees’ organizational identification and perceived supervisor moral decoupling. Additionally, this study discusses the moderating effect of employee bottom-line mentality on these relationships. We test this two-path model using a sample of MBA students in China. The results indicate that both organizational identification and perceived supervisor moral decoupling mediate the relationship between CSR and cheating, and employee bottom-line mentality moderates the effect of CSR on perceived supervisor moral decoupling. Specifically, for employees low in bottom-line mentality, CSR has a significantly negative impact on perceived supervisor moral decoupling, but the same relationship is insignificant for employees with a strong bottom-line mentality. Overall, our results uncover the relationship between CSR and employee cheating and extend the understanding of the influence of CSR on employees.

## Introduction

Corporate social responsibility (CSR) has attracted huge attention (Aguinis and Glavas, 2012; Matten and Moon, 2020), especially after societal inequalities become a serious challenge again due to the COVID-19 pandemic (Bapuji et al., 2020). CSR is defined as “context-specific organizational actions and policies that take into account stakeholders’ expectations and the triple bottom line of economic, social, and environmental performance” (Aguinis, 2011, p. 858). Through CSR initiatives, organizations take the interests of a wide range of stakeholders into account and remark their pro-social concerns and ethics (Glavas, 2016; Bapuji et al., 2020). As employees’ subjective perception of CSR is likely to determine their motivation and behavior in support of the organization (e.g., Rupp et al., 2013b; De Roeck and Maon, 2018), recent research attention has been paid to examine the employee-level outcomes of CSR (Aguinis and Glavas, 2012; De Roeck et al., 2016). Most studies on the consequences of CSR focus on its influences on beneficial attitudes and behavior of employees (e.g., organizational identification, organizational commitment, organizational citizenship behavior, and social responsible behaviors) (e.g., Glavas, 2016; Farooq et al., 2017; De Roeck and Maon, 2018; Wang et al., 2019). Unfortunately, few studies have examined how employee perception of CSR can influence their unethical behavior, such as cheating or deviance behavior, as well as uncover the mechanism underlying the relationship between employee perception of CSR and unethical behavior.

However, unethical behavior and fraud increase at a climbing rate (PwC PwC’s Global Economic Crime and Fraud Survey 2020) and incur potential loss for companies (Baker et al., 2019; Browning et al., 2019). Scholars strive to understand the causes of unethical behavior and find ways to counteract it (De Cremer and Moore, 2020; Veetikazhi et al., 2020). Recent ethical research indicates that (un)ethical behavior depends on the interaction between the individual and his or her environment (Kish-Gephart et al., 2010; De Cremer and Moore, 2020). For instance, Chen et al. (2021) find that the stretch goals of employees induce unethical behavior especially when employees consider the environment as competitive; Simha and Parboteeah (2020) suggest that the influence of individual personalities (e.g., agreeableness and conscientiousness) on their justification of unethical behavior relies on the national culture (e.g., institutional collectivism and humane orientation). When it comes to the unethical behavior fighting approach, studies have shown that the organization’s ethical infrastructure can exert a critical impact (Kaptein, 2011; Thornton and Rupp, 2016; Constandt et al., 2019; Veetikazhi et al., 2020).

In line with this, prior work anticipates that CSR can reduce unethical behavior. However, the results of these limited studies are conflicting. For example, Evans et al. (2010) show that perceived corporate citizenship negatively affects employee deviance through organizational cynicism. Meanwhile, CSR also results in corporate social irresponsibility (CSIR) because commitment to CSR endow top managers with moral license, especially when they have high levels of moral identity symbolization (Ormiston and Wong, 2012). The lack of consistent evidence on the effects of CSR on unethical behavior indicates the need for further research. Given the prevalence and huge costs of employee cheating (Mitchell et al., 2018; Vadera and Pathki, 2021), this study takes cheating as a typical form of unethical behavior and tries to determine when and how CSR affects employee cheating.

Because CSR signifies the concerns of organizations for social welfare, extant research suggests that it is an important source of social information that employees may utilize to interpret the morality of their organization and their supervisor (Rupp et al., 2013a; Jones et al., 2014; Gao and He, 2017). Accordingly, by using social information processing theory as an overarching framework, we propose that there are two pathways through which CSR impacts employee cheating. Social information processing theory states that individuals develop attitudes and behavior resulting from the processing of social information deriving from the social environment where they embed in Salancik and Pfeffer (1978), Vlachos et al. (2014), De Roeck and Maon (2018). CSR signals organizational concerns about ethics and social welfare (Rupp and Mallory, 2015; Ong et al., 2018), which results in the organization being an attractive entity for employees to identify with (Farooq et al., 2017), and employees, in turn, discouraged from cheating. Different from genuine care of the organization deriving from organizational identification, we posit that CSR can also suppress cheating for fear of being punished by their supervisors. Through CSR initiatives, organizations send clear information cues about the importance of social welfare (Rupp and Mallory, 2015; Gao and He, 2017; De Roeck and Maon, 2018). Learning from CSR signals, employees are likely to expect that their leaders will imitate organization’s ethical values (Gao and He, 2017) and give priority over morality in addition to performance. We therefore suggest that supervisors in organizations that are active in CSR initiatives will be inferred and perceived by employees as having a low level of moral decoupling. Moral decoupling is defined as a moral reasoning process whereby individuals “dissociate the judgments of morality from the judgments of performance” (Bhattacharjee et al., 2013, p. 1168). Under the supervision of leaders who are perceived as not having moral decoupled, employees are more likely to stop cheating because they are afraid of the negative evaluation or even punishment given by their supervisors.

Although CSR acts as social information to affect employees’ cheating, the significance of CSR for employees to develop attitudes and behavior depends on their levels of CSR sensitivity (Ong et al., 2018). Our theorizing is accordingly further enriched by exploring how employee differences in bottom-line mentality moderate the impact of CSR on organizational identification, perceived supervisor moral decoupling, and employee cheating. Bottom-line mentality refers to one-dimensional thinking centered on bottom-line outcomes (Greenbaum et al., 2012), which could influence employees’ sensitivity to CSR activities. Since employees with a high bottom-line mentality focus exclusively on bottom line and neglect other competing priorities (Greenbaum et al., 2012; Eissa et al., 2019), CSR signals become less important in the eyes of these employees and have less influence on the employees. Accordingly, we propose that the influence of CSR, which is exerted either on organizational identification or on perceived supervisor moral decoupling, will be attenuated when employees have a high level of bottom-line mentality, thus weakening the negative relationship between CSR and employee cheating.

To test our research model of the effect of CSR on employee cheating, we conduct a cross-sectional field study with a sample of MBA students in China. This research makes several contributions to the extant research on the impact of CSR at the employee level and the influence of employee bottom-line mentality. First, this study extends our understanding of the effects of CSR on unethical behavior by uncovering the mechanisms underlying CSR and employee cheating, a typical unethical behavior. Existing studies of CSR at the employee level have focused most on the effect of CSR on positive attitudes and behaviors (Evans et al., 2011; Farooq et al., 2017), but paid little attention to its impact on unethical behavior. Based on social information processing theory, we complement the extant research on CSR by indicating that CSR could reduce cheating *via* organizational identification and perceived supervisor moral decoupling. Second, by exploring employee bottom-line mentality as a boundary condition of the relationship between CSR and employee cheating *via* perceived supervisor moral decoupling, we, to some extent, reconcile the conflicting past findings on the impact of CSR on employee unethical behavior. As previous studies have shown conflicting results on the relationship between CSR and employee unethical behavior, our study makes an initial attempt to integrate those conflicting findings by showing that CSR affects employee cheating behavior less significantly when employee bottom-line mentality is low than high. Furthermore, we also contribute to the bottom-line mentality literature by showing moderating effects of employee bottom-line mentality on the association between CSR and cheating through perceived supervisor moral decoupling. Prior bottom-line mentality studies focus exclusively on the impact of supervisor bottom-line mentality; recent researchers begin to advocate more attention on employee bottom-line mentality (Eissa et al., 2019; Quade et al., 2020). In response to their calls, we introduce employee bottom-line mentality into CSR literature and find that employee bottom-line mentality affects their sensitivity to CSR and shapes the indirect relationship from CSR to cheating *via* perceived supervisor moral decoupling. Doing so expands our understanding of the outcomes of employee bottom-line mentality.

## Hypothesis Development

### CSR and Organizational Identification

Since Aguinis and Glavas (2012) garnered interests in the microfoundations of CSR, the following studies have endeavored to examine the effects of employees’ perception of CSR on employee attitudes and behavior (De Roeck et al., 2016; Paruzel et al., 2021; Zhao et al., 2022). Organizational identification has been the most widely used theoretical mechanism to explain the effect of CSR at the individual level (Gond et al., 2017; Zhao et al., 2022), and it has also been found to be an important mediator between CSR and employee-related outcomes such as commitment, job satisfaction, and OCB (Farooq O. et al., 2014; Farooq et al., 2017; El Akremi et al., 2018; Paruzel et al., 2021). Organizational identification, defined as “perceived oneness with an organization and the experience of the organization’s successes and failures as one’s own” (Mael and Ashforth, 1992, p. 103), creates a bond between employees and their organizations such that organizations can satisfy employees’ desires for belongness and self-enhancement (Dutton et al., 1994).

According to the self-enhancement account of social identification, employees are more likely to identify with organizations that have a distinctively positive image (Ashforth and Mael, 1989; Dutton et al., 1994; Carroll, 1995). The investment in CSR reflects that the organization takes interests of multiple stakeholders into account and make a significant contribution to social welfare (Aguinis and Glavas, 2012; Farooq et al., 2017), thereby conveying a responsible and positive image for employees to promote their organizational identification (Jones et al., 2014; De Roeck et al., 2016; Abdullah et al., 2017). Besides that, recent research indicates that ethical climates are directly related to organizational identification (DeConinck, 2011; Pagliaro et al., 2018). In fact, employees prefer to identify with organizations that are considered as moral (Ellemers et al., 2013; van Prooijen and Ellemers, 2015). CSR subsumes pro-social concerns of the organization to various stakeholders and shows the employees the morality and ethics of their organization (Jones et al., 2014; Baskentli et al., 2019), which are germane to forming a strong ethical climate, and then facilitate employees’ organizational identification (Pagliaro et al., 2018; Kim and Choi, 2021). In sum, CSR activities reflect the morality of the organization and distinguish it positively from organizations that do not invest in CSR, making it attractive for employees to identify with.

Therefore, the hypothesis is as follows:

*Hypothesis 1a: CSR positively influences employees’ organizational identification.*

We further propose that CSR negatively affects employee cheating behavior through organizational identification. As a common unethical behavior in organization, cheating is repeatedly found to be related with high performance pressure and competition (e.g., Mitchell et al., 2018; Vadera and Pathki, 2021). Accordingly, employee cheating might increase rapidly amid the COVID-19 pandemic and pose serious problems for the organization, since huge pressure and job insecurity confront employees (Fu et al., 2021; Lin W. et al., 2021). Organizations need approaches to fight against employee cheating. We expect that organizational identification can prevent employees from cheating.

Employees who identify with their organization define themselves based on organization identity and image (Hogg and Terry, 2000). Organizational identification motivates employees to adhere to organizational norms (Dutton et al., 1994) and devote their efforts for the sake of the organization (Ashforth and Mael, 1989). Accordingly, we posit that organizational identification can reduce cheating behavior that focuses exclusively on the personal interests without consideration of the collective well-being (Mitchell et al., 2018; Hillebrandt and Barclay, 2020). Furthermore, since employees high in organizational identification tend to derive part of their identities from the organization and stick to organizational norms (Dutton et al., 1994; Ellemers et al., 2013), they are likely to follow ethics that are elucidated and highlighted by CSR. To sum up, as CSR signals organization’s concerns on the well-being of various stakeholders and establishes an ethical image for the organization, CSR promotes employees to identify with their organizations and comply with organizational ethical image, which in turn discourages employees’ unethical cheating behavior.

Therefore, our next hypothesis is as follows:

*Hypothesis 1b: Organizational identification mediates the relationship between CSR and cheating behavior.*

### CSR and Perceived Supervisor Moral Decoupling

We further predict that employees use CSR as social information to infer their supervisors’ moral decoupling. According to Bhattacharjee et al. (2013), moral decoupling is distinct from other moral reasoning strategies such as moral rationalization (Bhattacharjee et al., 2013; Lee and Kwak, 2016). Individuals using moral rationalization tend to justify immoral actions and strive to reconstruct transgressions as less immoral than they are (Tsang, 2002; Bhattacharjee et al., 2013). Unlike moral rationalization, moral decoupling enables individuals to condemn transgressions in accordance with their moral standards, while also evaluating transgressors as high-performers (Bhattacharjee et al., 2013; Lee and Kwak, 2016). Hence, moral decoupling is more accessible than moral rationalization, especially when there is no vagueness regarding the morality of the behavior (Lee and Kwak, 2016; Chen et al., 2018). Given the clearly unethical nature of cheating (Mitchell et al., 2018; Hillebrandt and Barclay, 2020), the perceived supervisor moral decoupling by employees is expected to be of more relevance than moral rationalization, as it affects how supervisors are perceived to react to and evaluate cheating employees. We predict that CSR may function as social information and provide employees with valuable information for perceiving supervisor moral decoupling.

First, frequent CSR activities inform middle- and grassroots-level supervisors that the organization and their top management teams place emphasis on pro-social values and moral actions besides economic performance (Aguinis, 2011; Gao and He, 2017). Accordingly, middle- and grassroots-level supervisors are more likely to simultaneously emphasize economic and moral interests rather than emphasize only one aspect, leading them to have a weak intention of moral decoupling.

Furthermore, as supervisors vary in the weights giving to performance and morality, their moral decisions and actions might be ambiguous and ill-defined to interpret (Reynolds, 2003). Employees need clues to help them infer supervisors’ moral decoupling (Fehr et al., 2019). CSR involves an explicit emphasis of organizational focus from purely economic to a range of stakeholders (e.g., community, consumers, charity, etc.) (Aguinis and Glavas, 2012; Rupp and Mallory, 2015; Farooq et al., 2017). Employees can draw an inference from CSR initiatives that the organization and its management team value social and ethical commitments in addition to financial performance (Rupp et al., 2006; Evans et al., 2010; Rupp, 2011), and be more likely to perceive their supervisors as simultaneously interested in ethics and performance and having a low tendency toward moral decoupling. In support of this argument, Gao and He (2017) found that CSR could shape the level of ethical leadership perceived by employees.

To sum up, it can be said that the ethical considerations and moral information contained in CSR not only lead supervisors to demonstrate the low tendency toward moral decoupling but also shape the perception of employees that their supervisor does not evaluate performance independent of his or her morality. Thus, employee perception of CSR is negatively related to perceived supervisor moral decoupling.

*Hypothesis 2a: CSR negatively influences perceived supervisor moral decoupling.*

Despite the unethical nature of cheating, the unethical behavior has not always been rejected or even poorly rated by the organization or the supervisor (e.g., Quade et al., 2017; Zhu et al., 2021). Recent studies have indicated that supervisors can tolerant unethical employees, especially when supervisors are moral decoupling (Zhang G. et al., 2021). With this in mind, we attempt to explore the influence of the perceived supervisor moral decoupling on employee cheating in this study.

Moral decoupling was first examined in the marketing literature, which has repeatedly demonstrated the influence of moral decoupling on continuing support for unethical public figures or immoral purchases (e.g., buying counterfeits) (Bhattacharjee et al., 2013; Orth et al., 2019). Recent studies have extended moral decoupling to the organizational context and explored the influence of supervisor moral decoupling. Fehr et al. (2019) find that when supervisors are perceived as high in moral decoupling, employee concerns about wrongdoing dissipate, leading them to imitate their supervisors’ unethical pro-organizational behavior. Similarly, we argue that the perceived supervisor moral decoupling by employees is likely to arouse cheating behavior, as it ensures that employees are not negatively appraised for cheating.

Employees’ cheating behavior is largely driven by self-interest (Vadera and Pathki, 2021; Zhu et al., 2021). However, negative ratings or penalties for cheating reduce the benefits of cheating and prevent employees from cheating. In line with this, we hypothesize that cheating is more beneficial when employees perceive their supervisor as moral decoupling. Supervisors perceived high in moral decoupling are prone to separate performance appraisals from moral judgments and to condone cheating. In such contexts, employees tend to believe that cheating is unlikely to result in negative performance evaluation, and consequently, they cheat. Conversely, when employees perceive their supervisors to have low levels of moral decoupling, they worry that unethical cheating behavior will undermine their performance appraisal, thus reducing the benefits of cheating. Thus, employees who perceive their supervisors as having low levels of moral decoupling are less disposed to cheat. In sum, we hypothesize that perceived supervisor moral decoupling positively influences employee cheating and further mediates the relationship between CSR and cheating.

*Hypothesis 2b: Perceived supervisor moral decoupling mediates the relationship between CSR and cheating behavior.*

### Moderating Effects of Employee Bottom-Line Mentality

Based on social information processing theory, we suggest that CSR reduces cheating, either through organizational identification, which tightly binds the organization fate with the individual destiny, or through fear of possibly negative evaluations by supervisors who are perceived as less morally decoupled. This perspective also recognizes that employees vary in the utilization and interpretation of social cues because of their personality difference (e.g., Young et al., 2009). Combining this argument with previous CSR studies (Ong et al., 2018), we further predict that these relationships are moderated by employee bottom-line mentality.

Bottom-line mentality, which is “one-dimensional thinking that revolves around securing bottom-line outcomes to the neglect of competing priorities” (Greenbaum et al., 2012, p. 344), contributes to the survival of the organization and the financial performance (Cushen, 2013). Research suggests that employees led by supervisors with the bottom-line mentality feel obligated to the bottom line (Babalola et al., 2021) and mentally preoccupied with work (Babalola et al., 2020), thereby improving their performance (Zhang et al., 2020; Babalola et al., 2021). While bottom-line mentality can potentially benefit organizations, it has many negative implications on performance (Eissa et al., 2019; Quade et al., 2020; Lin Y. et al., 2021). For example, when supervisors are engrossed with solely bottom-line goals, their followers suffer from negative experience, such as insomnia, work-family conflict, and emotional exhaustion (Farasat et al., 2021; Quade et al., 2021; Wan et al., 2021). Supervisor bottom-line mentality also provokes unethical problems on the part of employees (Mesdaghinia et al., 2019; Babalola et al., 2021).

Although the influence of supervisor bottom-line mentality has drawn most attention, the research on employee bottom-line mentality, while in its emerging, shows that employee bottom-line mentality equally impacts employee attitudes and behavior (Eissa et al., 2019; Scrimpshire et al., 2021). Because employee bottom-line mentality entails employees with a tunnel vision, who focus exclusively on bottom line, and instigates a win-lose mindset in their minds, employee bottom-line mentality fuels knowledge hiding and social undermining (Greenbaum et al., 2012; Zhang Y. et al., 2021), and reduces organizational citizenship behavior toward coworkers (Eissa et al., 2019). In addition to its direct influence, employee bottom-line mentality makes employees willing to undermine colleagues when they watch their supervisor undermines others (Eissa et al., 2020). Moreover, Tai et al. (2021) find that women with a bottom-line mentality received more mistreatment because those women are more likely to be perceived as violating gender norms.

The burgeoning research on employee bottom-line mentality has accumulated valuable evidence, however, more work is still advocated to deepen our understanding of the influence of employee bottom-line mentality (Eissa et al., 2019; Quade et al., 2020). In response to such calls, this study proposes that employee bottom-line mentality will affect employees’ sensitivity and attitudes toward CSR, as employees with a high bottom-line mentality regard the bottom line as the only important thing and assign less weight to the information of CSR, and thus mitigate the influence of CSR on cheating behavior *via* organizational identification and perceived supervisor moral decoupling.

First, as argued above, employees tend to identify with organizations that engage in CSR because CSR reflects an organization’s social concerns and virtues, improving the reputation of the organization and satisfying individual desire for self-enhancement (De Roeck et al., 2016; Farooq et al., 2017). However, these positive effects of CSR on organizational identification might not occur if employees do not regard CSR highly or even consider CSR as a burden on the organization. As employee bottom-line mentality activates one-dimensional thinking about bottom-line outcomes irrespective of other priorities (Greenbaum et al., 2012; Quade et al., 2020), the informational values of CSR, reflecting a typical kind of moral and pro-social consideration of the corporate, will be underestimated and less salient in the eyes of employees with a bottom-line mentality. In addition, such employees might consider the investment in CSR as a waste of organizational resources that otherwise could be used to secure the bottom line. Hence, we suggest that employees with a high bottom-line mentality are less likely to be affected by CSR and not to feel pride to identify with the organization because of its CSR.

By contrast, employees having a weak bottom-line mentality are more likely to consider the interests of various stakeholders than those with a high bottom-line mentality and attach great importance to CSR to determine their attitudes and behavior. For these employees, CSR is a useful way of balancing the interests of multiple stakeholders and they appreciate it. Therefore, the positive relationship between CSR and organizational identification remains significant when employee bottom-line mentality is weak.

The hypothesis is summarized as follows:

*Hypothesis 3a: Employee bottom-line mentality moderates the relationship between CSR and organizational identification, such that the positive relationship between CSR and organizational identification is stronger when employee bottom-line mentality is weak.*

We also propose that employee bottom-line mentality moderates the relationship between CSR and perceived supervisor moral decoupling by employees. Given the explicitly moral relevance of CSR initiatives, we suggest above that employees attend to the information of CSR to infer their supervisor’s moral decoupling. However, the information of CSR might become less salient for employees with a high level of bottom-line mentality than for those with a low level and less useful for the former in shaping the perception of supervisor moral decoupling.

Specifically, strong employee bottom-line mentality causes individuals to adopt one-dimensional thinking and to generate an exclusive focus on the bottom line (Greenbaum et al., 2012; Eissa et al., 2019; Quade et al., 2020). Studies show that such a one-dimension mentality activates a win–lose mindset and stimulates the self-interested concern of the employee (Babalola et al., 2020). Employees with a high bottom-line mentality thus rely more on information relevant to their interests and the bottom line than on other information, such as how stakeholders are treated shown by organization’s CSR activities. Information about CSR activities becomes less salient for employees with high levels of bottom-line mentality, who consequently less intend to rely on the extent of CSR activities to infer supervisor moral decoupling. Accordingly, the effects of CSR on perceived supervisor moral decoupling could be mitigated under high levels of employee bottom-line mentality.

Contrary to employees having high levels of bottom-line mentality, employees low in bottom-line mentality are more likely to strive for multiple objectives. Such employees are disposed to focus on both financial performance and social responsibility. They are likely to take into account organization’s CSR initiatives and be influenced by CSR. Employees who have a low level of bottom-line mentality will accordingly use the extent of CSR initiatives to deduce supervisor moral decoupling. Therefore, we expect a more significant relationship between CSR and perceived supervisor moral decoupling among employees with a weak bottom-line mentality.

The hypothesis is summarized as follows:

*Hypothesis 3b: Employee bottom-line mentality moderates the relationship between CSR and perceived supervisor moral decoupling, such that the negative relationship between CSR and perceived supervisor moral decoupling is weaker when employee bottom-line mentality is high.*

Combining our hypothesis on the negative effects of CSR on cheating behavior *via* organizational identification and perceived supervisor moral decoupling with our hypothesis on the moderating effects of employee bottom-line mentality, we propose that employee bottom-line mentality will further moderate these indirect relationships.

*Hypothesis 4a: Employee bottom-line mentality moderates the indirect relationship between CSR and cheating *via* organizational identification, such that the indirect relationship is stronger when employee bottom-line mentality is weak.*

*Hypothesis 4b: Employee bottom-line mentality moderates the indirect relationship between CSR and cheating *via* perceived supervisor moral decoupling, such that the indirect relationship is stronger when employee bottom-line mentality is high.*

**FIGURE 1:**
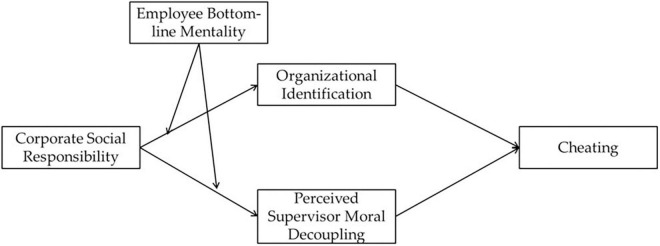
Conceptual model.

The conceptual model of this study is summarized in Figure 1.

## Materials and Methods

### Sample and Procedure

We obtained data from full-time employees who were participating in part-time MBA programs at a famous university in China. MBA students from such business programs have a variety of occupational and industrial backgrounds. Because MBA students are usually concerned about their career prospects and whether they could get fair treatment from their employers, they are more likely to be interested in organizations’ CSR activities. After gaining their approval, we sent the participants a link to our questionnaire. They completed the questionnaire at their convenience over the following 3 days. We sent 207 questionnaires to these MBA students, and a total of 197 MBA students participated in the survey and successfully returned their questionnaires.

The mean age of the participants was 30.60 years old (SD = 4.69), and 39% were male. They worked for the current company for an average of 6.09 years (SD = 3.73), and worked in various industries, including manufacturing industries (14%), service industries (70%), and others (16%). The participants also represented a wide range of organizational types (39% from state-owned companies, 43% from private enterprises, 8% from foreign companies, and the remaining from other companies) and occupations (28% working in HR/accounting departments, 21% in marketing department, 11% in R&D, 6% in manufacturing, and the rest in other departments). Additionally, the participants varied in their job rankings: junior staff (37%), front-line managers (39%), middle managers (20%), senior managers (3%), and others (1%).

### Measures

All of the measures were adopted from published papers and their validity has been confirmed in previous studies. We translated these measures into Chinese according to translation back-translation procedure (Brislin, 1980). Unless otherwise mentioned, all of the ratings were based on the same 7-point Likert scale (from 1 = “Strongly disagree” to 7 = “Strongly agree”).

#### CSR

We measured CSR with 13 items adopted from Farooq et al. (2017). The scale was developed and tested by a series of Farooq and his colleagues’ work (Farooq M. et al., 2014; Farooq O. et al., 2014), which initially aimed to depict employee’s perception of their company’s CSR and has been used to capture employee’s perception of their company’s CSR especially in an Asian context (Hu et al., 2020; Kim et al., 2021). Among the 13 items, four items focus on CSR to environment (e.g., “Our company implements special programs to minimize its negative impact on the natural environment”), three items focus on CSR to community (e.g., “My company contributes to the campaigns and projects that promote the well-being of the society”), three items focus on CSR to consumers (e.g., “My company provides full and accurate information about its products to its customers”), and three items focus on CSR to internal employees (e.g., “Our company primarily concerns with employees’ needs and wants”). The Cronbach’s α of this scale was 0.92, above the acceptable standards (>0.70).

#### Employee Bottom-Line Mentality

Employee bottom-line mentality was measured using the four-item scale from Greenbaum et al. (2012). As Greenbaum et al. (2012) developed the bottom-line mentality scale for both supervisors and subordinates, based on our research objective, we adopted the employee version of the bottom-line mentality scale. The four items were “I am solely concerned with meeting the bottom line,” “I only care about the business,” “I treat the bottom line as more important than anything else,” and “I care more about profits than employee well-being.” The same scale has also been used to measure the bottom-line mentality of employees by other studies (e.g., Eissa et al., 2020; Scrimpshire et al., 2021; Tai et al., 2021), providing further evidence for the validity of this scale. The Cronbach’s α of this scale was 0.85.

#### Organizational Identification

We measured organizational identification with Mael and Ashforth’s (1992) six-item scale, which is the most frequently used scale for this variable (e.g., Tangirala and Ramanujam, 2008). Sample items were “When someone criticizes my company, it feels like a personal insult” and “When I talk about this company, I usually say “we” rather than “they.” The Cronbach’s α was 0.88.

#### Perceived Supervisor Moral Decoupling

The five-item scale used by Fehr et al. (2019) was adopted to measure perceived supervisor moral decoupling by employees. Fehr et al. adapted the scale from Bhattacharjee et al. (2013) and modified the original scale to assess to the extent to which employees perceived their supervisors’ moral decoupling. A sample item was “An employee’s unethical actions do not change my supervisor’s assessments of that employee’s performance on work tasks.” The Cronbach’s α was 0.89.

#### Cheating

We measured cheating behavior using a seven-item scale developed by Mitchell et al. (2018). Before measuring cheating behavior, we conducted several interviews with employees in China to assure the validity of the scale. According to our interview, employees mentioned several typical cheating behaviors in their workplace, such as “pretend to work while they are cyberloafing,” “exaggerate the quality of the work when he or she knows this is not true,” “fabricate reasons for the delay in work,” “make up excuses for not attending job-related activities especially when there is no compulsory to attend,” “lie about the workload and work schedule in order not to take on more work,” etc., The interview showed that the seven-item scale still largely covered the typical cheating behavior. The scale has been used by other studies in China (e.g., Sun et al., 2019; Men et al., 2021). The participants were asked to assess how often they engage in each cheating behavior. Examples were “Made it look like you were working when you were not” and “Came in late and didn’t report it.” The response format ranged from 1 “never” to 7 “always.” The Cronbach’s α was 0.89.

#### Control Variables

Previous studies have considered the role of individual demographic characteristics in analyses of the effects of CSR on employees (Rupp and Mallory, 2015). Accordingly, we controlled for participants’ gender, age, educational level, job rank, and organizational tenure in our model, as these characteristics may affect how employees perceive CSR (Post et al., 2011; Wisse et al., 2018) and their unethical behavior (Loe et al., 2000; Mitchell et al., 2018). We measured job rank by asking participants to choose one of the following classifications: junior staff, frontline manager, middle manager, senior manager, or others.

We also controlled for several organization-level variables. In particular, state-owned enterprises (SOEs) usually need to assume more social responsibilities (Bai et al., 2006; Li and Zhang, 2010), which might affect how employees expect SOEs’ CSR and react to them. We thus added the ownership of organizations into the regression as a dummy variable based on whether the organization was state-owned or not. We further controlled for industry types of organizations because prior research suggested that corporate social performance varied widely from service sector to other sectors (e.g., Carballo-Penela and Castromán-Diz, 2015; Cai et al., 2016) and exerted different effects on employees’ perceptions of CSR (Vlachos et al., 2014; del Mar Miras-Rodríguez and Di Pietra, 2018). We then controlled for industry type of organizations as a dummy variable based on whether organizations were service sector or not.

Furthermore, because employees may rely on cues from their ethical leaders, as another significant social information source, to make sense of the environment and to interpret the moral standard of the organization (Zhang et al., 2018; Wadei et al., 2021), we controlled for ethical leadership perceived by employees in the following analysis using the 10-item scale developed by Brown et al. (2005). The Cronbach’s alpha of this scale was 0.94.

## Results

### Preliminary Analysis

Since we conducted a cross-sectional study and collected all the variables at the same time, we first ran Harman’s single-factor test to test the common method variance (CMV). The results showed that the first extracted factor explained only 26% of the total variance in the data, suggesting that CMV was not a significant threat to this study (Podsakoff and Organ, 1986).

### Confirmative Factor Analysis

We first conducted a set of confirmative factor analyses (CFA) to assess the divergent validity of the key variables (i.e., CSR, employee bottom-line mentality, organizational identification, perceived supervisor moral decoupling, and cheating). Because we focus more on the distinctiveness of the key constructs rather than the intercorrelations or the factor structure within constructs, we conducted the item parceling. Item parceling is also conducive to obtaining an optimal ratio of sample size to number of estimated indicators in CFA analysis (Little et al., 2002). Particularly, in line with previous studies (Grant and Berry, 2011; Restubog et al., 2011), we used the dimensional scores to yield four parcels for CSR and used the item-to-construct-balance method (Little et al., 2002) to generate three parcels for employee bottom-line mentality, organizational identification, perceived supervisor moral decoupling, and cheating. The hypothesized five-factor model revealed a good fit: χ^2^ = 206.27, *df* = 94, comparative fit index [CFI] = 0.95, standardized root mean square residual [SRMR] = 0.07, and root mean square error of approximation [RMSEA] = 0.08. In addition, the five-factor model had a better fit than any of the four-factor model (see Table 1) and the one-factor model (χ^2^ = 1521.21, *df* = 104, CFI = 0.34, TLI = 0.23, SRMR = 0.20, RMSEA = 0.26; Δχ^2^ = 1314.94, Δ*df* = 10, *p* < 0.001). The above results ensured the discriminant validity of our key variables.

**TABLE 1:** Model fit results for confirmative factor analysis.

Models	χ^2^	*df*	Δχ^2^/Δ*df*	*SRMR*	*RMSEA*	*CFI*	*TLI*
Hypothesized five-factor model	206.27	94		0.07	0.08	0.95	0.93
Four-factor model: Combining CSR and employee bottom-line mentality	584.46	98	94.55***	0.17	0.16	0.77	0.72
Four-factor model: Combining CSR and organizational identification	494.08	98	71.95***	0.09	0.14	0.81	0.77
Four-factor model: Combining CSR and perceived supervisor moral decoupling	615.55	98	102.32***	0.15	0.16	0.76	0.70
Four-factor model: Combining CSR and cheating	702.09	98	123.96***	0.18	0.18	0.72	0.65
Four-factor model: Combining employee bottom-line mentality and organizational identification	601.22	98	98.74***	0.15	0.16	0.76	0.71
Four-factor model: Combining employee bottom-line mentality and perceived supervisor moral decoupling	550.35	98	86.02***	0.12	0.15	0.79	0.74
Four-factor model: Combining employee bottom-line mentality and cheating	436.47	98	57.55***	0.10	0.13	0.84	0.81
Four-factor model: Combining organizational identification and perceived supervisor moral decoupling	609.03	98	100.69***	0.15	0.16	0.76	0.71
Four-factor model: Combining organizational identification and cheating	665.79	98	114.88***	0.16	0.17	0.73	0.67
Four-factor model: Combining perceived supervisor moral decoupling and cheating	639.58	98	108.33***	0.13	0.17	0.75	0.69

*N = 197. △ is the change relative to the hypothesized five-factor model. CFI = comparative fit index; TLI = Tucker-Lewis index; SRMR = standardized root mean-square residual; RMSEA = root mean squared error of approximation.*

***p < 0.001.

### Descriptive Statistics and Correlation Metrics

The descriptive statistics and correlation matrixes for the main variables are shown in Table 2. As predicted, CSR was positively related to organizational identification (*r* = 0.41, *p* < 0.01) and negatively related to perceived supervisor moral decoupling (*r* = −0.26, *p* < 0.01) and cheating (*r* = −0.15, *p* < 0.05). In addition, organizational identification was negatively associated with cheating behavior (*r* = −0.24, *p* < 0.01), and perceived supervisor moral decoupling was positively associated with cheating behavior (*r* = 0.31, *p* < 0.01).

**TABLE 2:** Descriptive statistics and correlation matrix.

Variable	Mean	*SD*	1	2	3	4	5	6	7	8	9	10	11	12	13
1. Gender	0.61	0.49	–												
2. Age	30.60	4.69	−0.09	–											
3. Education	2.34	0.49	−0.07	0.07	–										
4. Job rank	1.92	0.88	−0.08	0.52**	0.09	–									
5. Tenure	6.09	3.73	0.04	0.54**	−0.08	0.15*	–								
6. Perceived ethical leadership	4.67	1.33	0.01	0.03	−0.03	−0.02	0.10	(0.94)							
7. SOE dummy	0.39	0.49	−0.23**	−0.05	−0.01	−0.21**	0.12	0.01	–						
8. Industry dummy	0.70	0.46	0.03	−0.08	0.15*	−0.04	−0.03	0.03	−0.05	–					
9. CSR	4.91	1.15	−0.06	0.22**	0.03	0.04	0.24**	0.39**	0.07	−0.11	(0.92)				
10. Employee bottom-line mentality	3.04	1.28	−0.14*	−0.05	0.04	−0.09	−0.01	−0.24**	0.14*	−0.06	−0.07	(0.85)			
11. Organizational identification	5.15	1.11	−0.03	0.17*	−0.01	0.08	0.20**	0.23**	0.01	−0.11	0.41**	−0.11	(0.88)		
12. Perceived supervisor moral decoupling	3.69	1.30	−0.01	−0.07	0.06	−0.01	−0.02	−0.37**	0.03	0.05	−0.26**	0.37**	−0.21**	(0.89)	
13. Cheating	2.27	1.16	−0.13^†^	−0.18*	0.14*	−0.21**	−0.05	−0.07	0.12	0.07	−0.15*	0.48**	−0.24**	0.31**	(0.89)

*N = 197. The number in the parentheses are the Cronbach’s α coefficients.*

*CSR = corporate social responsibility. For gender, 0 = male; 1 = female. For Education, 1 = high school degree and the below, 2 = bachelor’s degree; 3 = postgraduate degree and the above. For job rank, 1 = frontline employees, 2 = grassroots managers; 3 = middle managers; 4 = senior managers; 5 = others. SOE dummy: 0 = non-state-owned companies (SOEs); 1 = state-owned companies (SOEs). Industry dummy: 0 = non-service sector; 1 = service sector.*

**, *, and ^†^ denote statistical significance at the 1, 5, and 10% levels, respectively.

### Hypotheses Testing

The regression results are summarized in Table 3. All of the variables were centered before entering the regression. Hypothesis 1 predicts a positive relationship between CSR and organizational identification. After entering the control variables, Model 2 in Table 3 indicated that CSR was positively associated with organizational identification (*b* = 0.31, *p* < 0.01), supporting Hypothesis 1a. Hypothesis 1b predicts that organizational identification mediates the relationship between CSR and cheating behavior. As shown in Model 9, organizational identification was negatively related to cheating behavior (*b* = −0.15, *p* < 0.05). To further test the indirect effect of CSR on cheating behavior *via* organizational identification, we conducted the bootstrapping estimations of indirect effects using PROCESS macro for SPSS (Preacher and Hayes, 2004; Preacher et al., 2007). The results of 5000 resamples, shown in Table 4, confirmed the significant indirect relationship between CSR and cheating behavior through organizational identification (indirect effect = −0.05, boot SE = 0.03, 95% CI [−0.102, −0.003] excluding zero). Hypothesis 1b was therefore supported.

**TABLE 3:** Regression results.

Variables	DV = Organizational identification			DV = Perceived supervisor moral decoupling			DV = Cheating		
	M1	M2	M3	M4	M5	M6	M7	M8	M9
1. Gender	−0.07	−0.05	−0.05	−0.00	0.08	−0.01	−0.30^†^	−0.18	−0.20
2. Age	0.04*	0.03	0.03	−0.02	−0.01	−0.02	−0.05*	−0.03	−0.02
3. Education	−0.01	−0.04	−0.04	0.14	0.12	0.09	0.39*	0.36*	0.33*
4. Job rank	−0.04	−0.02	−0.02	0.03	0.04	0.08	−0.19^†^	−0.17^†^	−0.17^†^
5. Tenure	0.02	0.01	0.01	0.02	0.02	0.03	0.03	0.02	0.02
6. Perceived ethical leadership	0.18**	0.06	0.06	−0.36**	−0.24**	−0.25**	−0.06	0.08	0.12*
7. SOE dummy	−0.03	−0.03	−0.03	0.08	0.02	−0.04	0.10	0.01	0.00
8. Industry dummy	0.23	−0.16	−0.15	0.13	0.15	0.09	0.06	0.10	0.06
7. CSR		0.31**	0.31**		−0.16*	−0.15^†^		−0.15*	−0.08
8. Employee bottom-line mentality		−0.06	−0.06		0.31**	0.30**		0.41**	0.37**
9. CSR × Employee bottom-line mentality			−0.01			0.14**			
10. Organizational identification									−0.15*
11. Perceived supervisor moral decoupling									0.13*
***F***	3.27**	4.83**	4.37**	4.10**	6.03**	6.28**	3.26**	8.79**	8.42**
***R*** ^2^	0.12	0.21	0.21	0.15	0.24	0.27	0.12	0.32	0.35
Δ***R*^2^**		0.09**	0.00		0.09**	0.03**		0.20**	0.03**

*N = 197. Unstandardized coefficients are presented in the table.*

*CSR = corporate social responsibility. For gender, 0 = male; 1 = female. For Education, 1 = high school degree and the below, 2 = bachelor’s degree; 3 = postgraduate degree and the above. For job rank, 1 = frontline employees, 2 = grassroots managers; 3 = middle managers; 4 = senior managers; 5 = others. SOE dummy: 0 = non-state-owned companies (SOEs); 1 = state-owned companies (SOEs). Industry dummy: 0 = non-service sector; 1 = service sector.*

**, *, and ^†^ denote statistical significance at the 1, 5, and 10% levels, respectively.

**TABLE 4:** Bootstrapping results of mediation.

Mediation Model	Coefficient	Boot SE	95% CI
CSR → Organizational Identification → Cheating	−0.05	0.03	(−0.102, −0.003)
CSR → Perceived Supervisor Moral Decoupling → Cheating	−0.02	0.01	(−0.061, −0.001)

*PROCESS macro (Model 4). Bootstrapping = 5000. CSR = corporate social responsibility. SE = Standard error. CI = Confidential Interval.*

Hypothesis 2a predicted that CSR is negatively linked to perceived supervisor moral decoupling. Consistent with Hypothesis 2a, there was a significantly negative association between CSR and perceived supervisor moral decoupling (*b* = −0.16, *p* < 0.05). Hypothesis 2b further posited that perceived supervisor moral decoupling mediated the relationship between CSR and cheating. As shown by Model 9 in Table 3, perceived supervisor moral decoupling was positively related to cheating (*b* = 0.13, *p* < 0.05). The bootstrapping results further corroborated the significant indirect relationship between CSR and cheating behavior through perceived supervisor moral decoupling. The 95% confidential interval (CI), excluding zero (indirect effect = −0.02, boot SE = 0.01, 95% CI [−0.061, −0.001]). Hypothesis 2b was supported.

Hypotheses 3a and 3b stated that employee bottom-line mentality could moderate the relationships between CSR on organizational identification and perceived supervisor moral decoupling. Model 3 in Table 3 showed that the interaction term of CSR and employee bottom-line mentality did not exert a significant effect on organizational identification (*b* = 0.01, *ns.*). Hypothesis 3a was not supported. Meanwhile, employee bottom-line mentality significantly moderated the relationship between CSR and perceived supervisor moral decoupling (*b* = 0.14, *p* < 0.01), and the interaction term of CSR and employee bottom-line mentality produced a significant increase in explained variance for perceived supervisor moral decoupling (Δ*R*^2^ = 0.03, *p* < 0.01).

**FIGURE 2:**
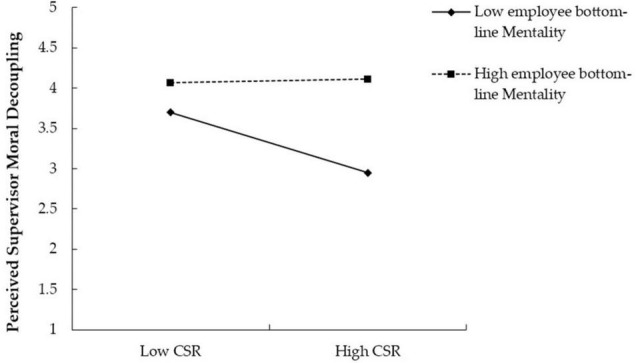
Moderation effects of employee bottom-line mentality on the relationship between CSR and perceived supervisor moral decoupling.

To provide further support for the moderating effects of employee bottom-line mentality, we plotted the moderated relationship in Figure 2 and conducted simple slope tests as described by Cohen et al. (2003). There was a significant relationship between CSR and perceived supervisor moral decoupling when employee bottom-line mentality was low (*b* = −0.33, *t* = −3.20, *p* < 0.01), whereas the same relationship became insignificant when employee bottom-line mentality was high (*b* = 0.02, *t* = 0.17, *ns.*). Hypothesis 3b was therefore supported.

According to Edwards and Lambert’s (2007) suggestion, in order to test the first-stage moderated mediation model proposed by H4a, we first need the interaction term of CSR and employee bottom-line mentality has a significant effect on organizational identification. Unfortunately, the relationship was not supported by our data. Therefore, the moderated mediation model posited by Hypothesis 4a could not be tested.

Hypothesis 4b posits that employee bottom-line mentality moderates the indirect effect of CSR on cheating through perceived supervisor moral decoupling. To test Hypothesis 4b, we analyzed the 95% bias-corrected confidential intervals for indirect effects at both high and low levels of employee bottom-line mentality based on 5000 bootstrap samples. As shown in Table 5, the indirect relationship between CSR and cheating behavior *via* perceived supervisor moral decoupling was significant (indirect effect = −0.08, boot *SE* = 0.04) and the 95% confidential interval was (−0.184, −0.025), excluding zero when employee bottom-line mentality was low; whereas the same indirect relationship became non-significant when employee bottom-line mentality was high (indirect effect = 0.00, boot *SE* = 0.03, 95% CI [−0.039, 0.069] including zero). The difference between the two indirect effects was 0.03 (boot *SE* = 0.02), and the 95% confidential interval was (0.008, 0.080), excluding zero, revealing a significant difference between the two indirect effects. Hypothesis 4b was therefore supported. We also ran analysis without any control variables^1^.

**TABLE 5:** Bootstrapping results of the moderated mediation model.

CSR → Perceived Supervisor Moral Decoupling → Cheating	Coefficient	Boot SE	95% CI
Low level of employee bottom-line mentality	−0.08	0.04	(−0.184, −0.025)
High level of employee bottom-line mentality	0.00	0.03	(−0.039, 0.069)
Difference	0.03	0.02	(0.008, 0.080)

*PROCESS macro (Model 7). Bootstrapping = 5000. CSR = corporate social responsibility. SE = standard error. CI = confidential interval.*

## Discussion

Corporate social responsibility has been linked to a wide range of positive behaviors and outcomes in previous studies (Farooq et al., 2017), but the influence of CSR on employees’ negative behavior has been largely neglected (Rupp and Mallory, 2015). This study uses social information processing theory to build a two-path model of the influence of CSR on employee cheating, and the results demonstrate that CSR has indirect effects on cheating *via* both organizational identification and perceived supervisor moral decoupling. Moreover, employee bottom-line mentality exerts a moderating effect on the relationship between CSR and perceived supervisor moral decoupling and the indirect effect of CSR on cheating behavior through perceived supervisor moral decoupling. Specifically, the indirect effect is significant when employee bottom-line mentality is low but becomes non-significant when employee bottom-line mentality is high. However, employee bottom-line mentality does not moderate either the relationship between CSR and organizational identification or the indirect relationship between CSR and cheating *via* organizational identification. Bottom-line mentality prompts employees to focus exclusively on the bottom line and give less weight to other dimensions (Mesdaghinia et al., 2019). We thus propose that employees with a high level of bottom-line mentality become less sensitive to the informational value of CSR. Meanwhile, as bottom-line mentality diverts employees’ attention only to bottom-line goals, employees who have a high level of bottom-line mentality might demand less for corporate social performance than those having low bottom-line mentality and accordingly reduce their expectation of corporate in terms of their investment in CSR. In such circumstances, while the information of CSR weighs less in the eyes of employees with a high level of bottom-line mentality, the level of CSR that they may feel satisfied with and lead to identify with their organization is also decreasing. The relationship between CSR and organizational identification might not be weakened by the high level of employee bottom-line mentality. Hence, employee bottom-line mentality neither significantly moderates the relationship between CSR and employee organization identification nor the indirect relationship between CSR and cheating *via* organizational identification. As research on employee bottom-line mentality is still in its infancy, we suggest further work to explore how employee bottom-line mentality affects employee concerns about CSR and organization’s morality while securing the financial performance.

### Theoretical Contributions

This study contributes to the literature in the following ways. First, by uncovering the significant influence of CSR on cheating *via* organizational identification and perceived supervisor moral decoupling, we broaden the understanding of CSR influence on unethical behavior. Extant studies have investigated mainly the link between CSR and positive OB attitudes and behavior (Gond et al., 2017; Gond and Moser, 2021) but yielded limited and conflicting understating of the effects of CSR on unethical behavior. As unethical behavior continues to be rampant and causes huge loss for organizations (Baker et al., 2019; Browning et al., 2019), we need to carefully examine the relationship between CSR and unethical behavior, in particular, when and how CSR is likely to reduce unethical behavior. Taking cheating as an example, this study demonstrates the negative effects of CSR on unethical behavior and uncovers the underlying mechanisms through which CSR affects unethical behavior. Specifically, we develop a two-path model showing that CSR can restrain cheating by increasing employees’ sincere concern for organizational interests (i.e., organizational identification) and by increasing their fear of being negatively appraised by supervisors (i.e., perceived supervisor moral decoupling).

Second, our findings further reconcile the current conflicting results of the impact CSR having on unethical behavior by identifying the boundary conditions under which employees are more or less likely to cheat based on CSR. While existing studies have paid limited attention to the relationship between CSR and employee unethical behavior, the limited results still fail to reach a consensus regarding the effects of CSR on employee unethical behavior (Evans et al., 2010; Ormiston and Wong, 2012). Based on social information processing theory and CSR sensitivity research, we find that employee bottom-line mentality moderates the relationship between CSR and cheating behavior such that the relationship becomes insignificant for employees who regard bottom-line outcomes as their primary goals, as CSR does not affect their perceptions of supervisor moral decoupling. Further studies are encouraged to explore the conditional factors that are likely to determine the relationship of CSR on employee unethical behavior and to provide a more comprehensive understanding of the effects of CSR on employee unethical behavior. Moreover, in the view of the explicit moral relevance of CSR (Evans et al., 2010; Barnett et al., 2020), existing studies that attempt to explore the conditional factors of CSR outcomes focus primarily on employees in terms of their moral dimensions such as moral identity (Evans et al., 2011; Rupp et al., 2013b; Gond et al., 2017). By exploring the moderating role of employee bottom-line mentality in the effects of CSR on cheating, our study adds nuances and reveals another type of individual difference, that is, the extent of employees’ focus on organizational financial performance, determining how employees appraise and react to CSR.

In addition, this study also contributes to the bottom-line mentality research by examining the moderating effects of employee bottom-line mentality on the association between CSR, perceived supervisor moral decoupling, and cheating. A growing body of research recently becomes interested in bottom-line mentality and explores its consequences (Greenbaum et al., 2012; Farasat et al., 2021). However, extant studies largely look at the impact of supervisor bottom-line mentality while neglecting the effects of employee bottom-line mentality. Since employee can develop the bottom-line mentality themselves (Eissa et al., 2019) and employee bottom-line mentality exerts the same or even more direct influences on employees’ attitudes and behavior than supervisor bottom-line mentality (e.g., Greenbaum et al., 2012; Zhang Y. et al., 2021), research calls for more attention on employee bottom-line mentality. In response to the call, this study finds that employee bottom-line mentality affects the sensitivity of employees toward CSR and the extent to which they rely on the degree of CSR to perceive supervisor moral decoupling and determine cheating, contributing to a better understanding of the influence of employee bottom-line mentality.

### Practical Implications

Our findings also have several practical implications. Most importantly, given the huge costs caused by cheating (Farasat et al., 2021; Vadera and Pathki, 2021), it is valuable to examine how and when CSR is likely to reduce cheating and other unethical behavior. Our study shows the process by which CSR affects cheating and underscores the importance of CSR in reducing employee cheating. We thus encourage organizations to inform employees of their CSR initiatives and lead employees to less cheat. Our study also shows that cheating can be significantly reduced when employees perceived their supervisor as having a low tendency toward moral decoupling. Accordingly, we suggest that it will help the organization to curb unethical behavior to educate their managers to balance the ethics and effectiveness. Finally, this study shows the detrimental impact of employee bottom-line mentality on employees’ responses to CSR. We find that employees with a high bottom-line mentality place less emphasis on CSR when deciding whether to cheat or not. In this vein, although bottom-line mentality enables the organization to secure the bottom-line outcomes, when the organization is trying to build an ethical environment, employee bottom-line mentality needs to be reduced, as employees with such mentality might not adhere to ethical norms.

### Limitations and Future Directions

Based on social information processing theory, we have developed a two-path model of the influence of CSR on employee cheating. We also examine the moderating effect of employee bottom-line mentality. Despite the contributions outlined above, this study has some limitations. First, as this study examines employees’ responses to and appraisal of CSR, all of the variables were reported by employees. Self-report questionnaires help us to understand employees’ opinions about CSR, but are subject to the common method variance (CMV) problem (Podsakoff et al., 2003). To ascertain the influence of CMV, we performed a CFA analysis of the one-factor model that combined five factors into one. The results showed that the one-factor model did not fit the data well. This suggests that CMV might not be a significant issue in this study. Moreover, as Siemsen et al. (2010) suggest, CMV actually mitigates rather than amplifies the interactional effect. Therefore, the interactional effect of CSR and employee bottom-line mentality identified in this study is still meaningful and worthy of attention because such effects cannot be the artifact of CMV. However, further research could re-examine our research model using a more rigorous research design.

Second, researchers suggest considering more individual differences and characteristics when we account for how employees respond to and evaluate CSR (Rupp and Mallory, 2015; Gond et al., 2017). This study investigates why employees with a high level of bottom-line mentality tend to neglect CSR information, and are thus less able to perceive supervisors’ moral decoupling and reduce cheating. As previous studies have repeatedly examined the main effects of CSR on employees (De Roeck and Maon, 2018), future studies could investigate the effects of different personal attributes on the CSR–employee relationship.

Third, we develop a two-path model based on social information processing theory to explain the influence of CSR on employee cheating. However, there could be other explanations of how CSR affects unethical behavior. For example, CSR has been found to create organizational trust according to social exchange theory (De Roeck and Maon, 2018). We further predict that the expected reciprocity between employees and the organization will reduce employee cheating and other forms of unethical behavior. In addition, Ormiston and Wong indicate a positive association between CSR and corporate social irresponsibility (CSIR) through moral licensing (Ormiston and Wong, 2012), indicating that CSR can sometimes harm organizations. Considering the limited research progress in the relationship between CSR and unethical behavior, we encourage further exploration of the relationship between CSR and unethical behavior, and especially studies to uncover CSR potentially detrimental impact.

## Conclusion

There are many studies of the relationship between CSR and employee positive attitudes and behavior, but when and how CSR influences unethical behavior has been largely ignored. As cheating is likely to cause immense losses to organizations, researchers are calling for more studies to prevent cheating. Using a sample of MBA students in China, our study examines the influence of CSR on employee cheating behavior. We first find that CSR can reduce cheating through organizational identification. Additionally, we hypothesize that employees use CSR to deduce their supervisors’ moral decoupling, which accounts for another pathway for CSR to reduce cheating. We further consider the moderating role of employee bottom-line mentality. Specifically, employee bottom-line mentality is found to moderate the effects of CSR on perceived supervisor moral decoupling such that the relationship becomes insignificant for employees high in bottom-line mentality. Considering the shortage of research about the influence of CSR on unethical behavior, this study first extends our understanding of how CSR inhibits cheating through organizational identification and perceived supervisor moral decoupling. The second contribution is to incorporate employee bottom-line mentality into CSR research and to demonstrate its significant role in determining how employees process information about CSR activities. Third, this study extends the understanding of bottom-line mentality by examining the moderating effect of employee bottom-line mentality on the relationship between CSR and cheating *via* perceived supervisor moral decoupling.

## Data Availability Statement

The original contributions presented in the study are included in the article/supplementary material, further inquiries can be directed to the corresponding author/s.

## Ethics Statement

The studies involving human participants were reviewed and approved by Academic Committee of Central University of Finance and Economics. The patients/participants provided their written informed consent to participate in this study.

## Author Contributions

KL was responsible for the research model development, research design, data analysis, and manuscript writing. ML was responsible for the data collection, data analysis, and manuscript writing. HZ was responsible for the manuscript revision. All authors have approved this research to be published.

## Conflict of Interest

The authors declare that the research was conducted in the absence of any commercial or financial relationships that could be construed as a potential conflict of interest.

## Publisher’s Note

All claims expressed in this article are solely those of the authors and do not necessarily represent those of their affiliated organizations, or those of the publisher, the editors and the reviewers. Any product that may be evaluated in this article, or claim that may be made by its manufacturer, is not guaranteed or endorsed by the publisher.

## Footnotes

1 We also ran the analysis without any control variables and the research hypotheses remained supported. CSR was positively related to organizational identification (*b* = 0.39, *p* < 0.01) and perceived supervisor moral decoupling (*b* = −0.29, *p* < 0.01). Organizational identification (*b* = −0.19, *p* < 0.01) and perceived supervisor moral decoupling (*b* = 0.24, *p* < 0.01) were again significantly related to employees’ cheating behavior. The interaction term between CSR and employee bottom-line mentality remained significant in predicting perceived supervisor moral decoupling (*b* = 0.12, *p* < 0.05) yet not significant in predicting organizational identification (b = −0.01, *ns.*).
